# Supplementary material for: Enhancer of zeste homolog 2 (Ezh2) is required in mouse Isl1-expressing progenitors for proper development of cardiac and skeletal hindlimb structures
Source: Biol Open. 2026 Apr 15;15(4):bio062550. doi: 10.1242/bio.062550 (PMC13133771; doi:10.1242/bio.062550)
Supplement: Supplementary information [file biolopen-15-062550-s1.pdf]

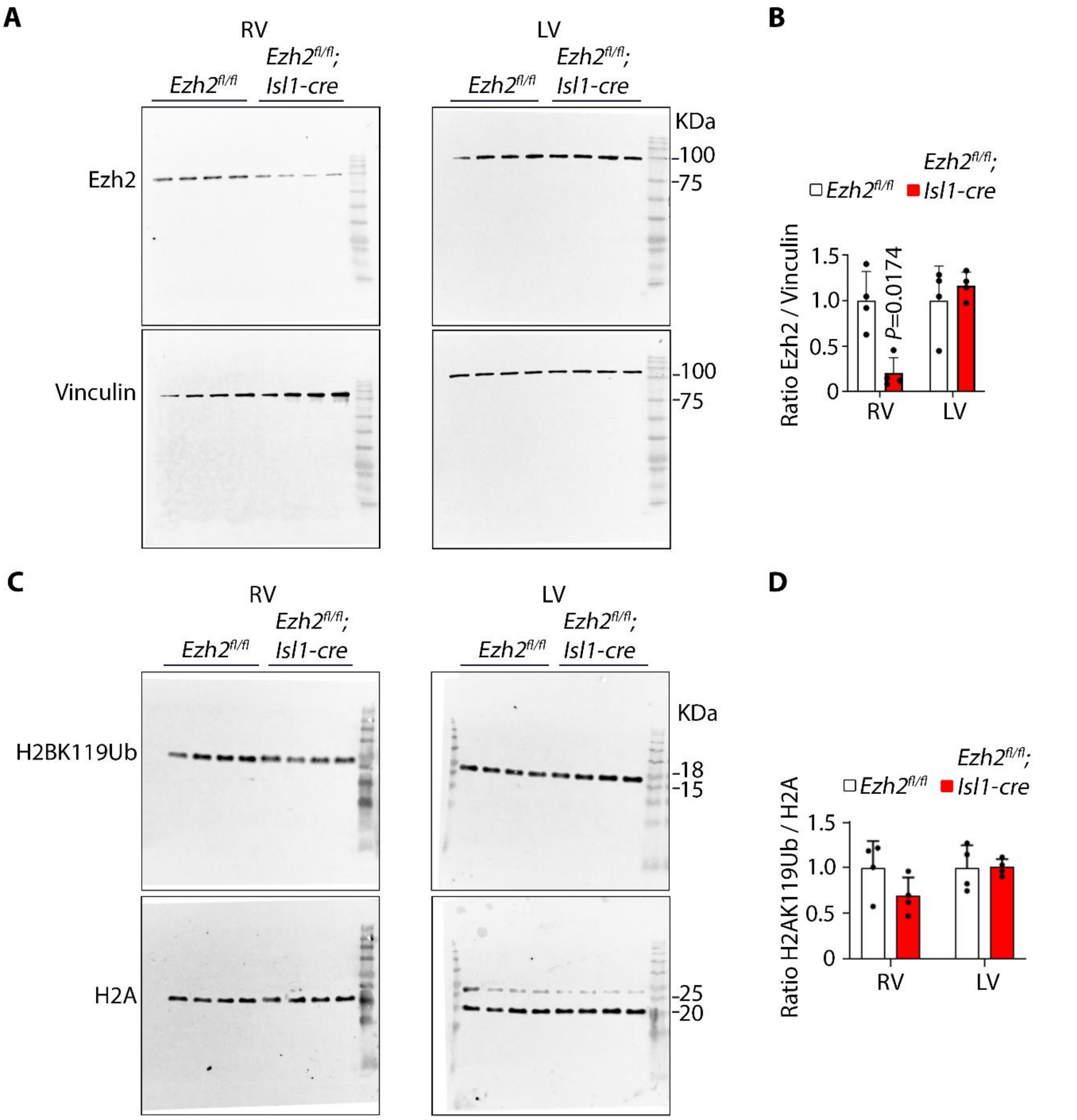

Fig. S1. Protein levels of Ezh2 and H2BK119ub in right and left ventricles of control and *Ezh2* mutant hearts at E16.5. (A, C) Western blots of Ezh2 and Vinculin (A), and H2BK119ub and H2A (C), in right ventricle (RV) and left ventricle (LV) lysates from control (*Ezh2$^{fl/fl}$*) and mutant (*Ezh2$^{fl/fl}$;Isl1-cre*) E16.5 hearts. Blots show protein lysates from 4 ventricles per genotype. (B, D) Quantification of Ezh2 normalized to Vinculin (B) and H2BK119ub normalized to H2A (D) in RV and LV lysates. Error bars denote mean±s.d. of 4 hearts per genotype, analyzed by two-tailed unpaired t-test with Welch's correction.

Biology Open • Supplementary information

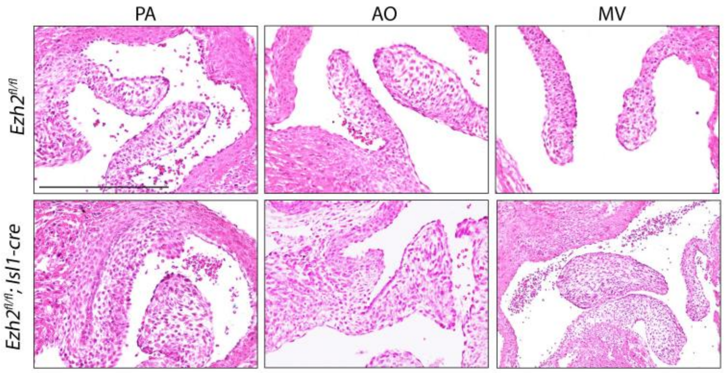

Fig. S2. Valve leaflet abnormalities in *Ezh2* mutant hearts at P0. Hematoxylin and eosin-stained sections of the pulmonary artery (PA), aortic valve region (AO), and mitral valve (MV) in control (*Ezh2$^{fl/fl}$*) and mutant (*Ezh2$^{fl/fl}$;Isl1-cre*) P0 hearts. Mutant hearts show thickened and irregular valve leaflets in the semilunar and atrioventricular valve regions compared with controls. Scale bar = 200 µm.

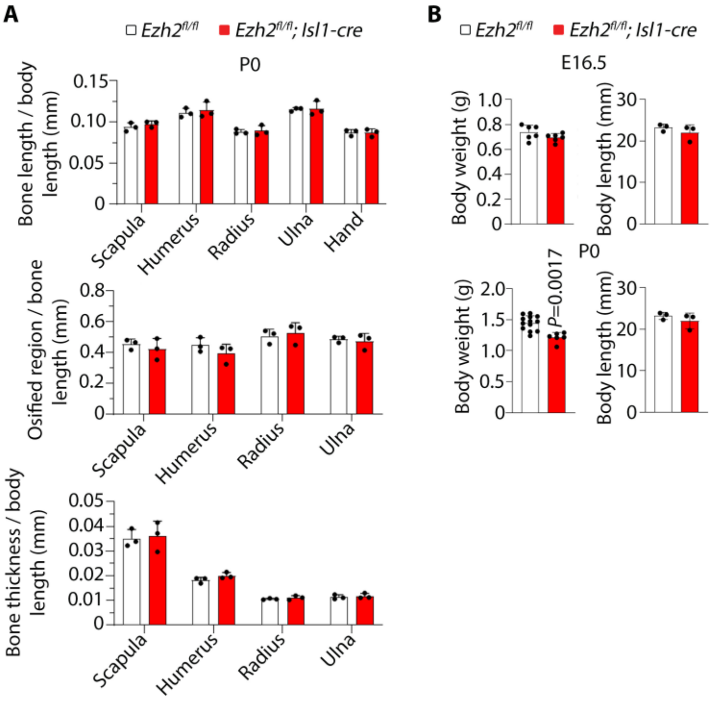

Fig. S3. Body size and forelimb skeletal measurements in control and *Ezh2* mutant embryos and pups. (A) Quantification of body weight and body length in control (*Ezh2$^{fl/fl}$*) and mutant (*Ezh2$^{fl/fl}$;Isl1-cre*) embryos at E16.5 and pups at P0. (B) Measurements of forelimb skeleton in P0 control and mutant pups, including bone length normalized to body length, extent of ossification, and bone thickness normalized to body length. Error bars denote mean±s.d. of 3 or 6 embryos or pups per genotype, analyzed by two-tailed unpaired t-test with Welch's correction.

Table S1. Primers and PCR conditions used for genotyping

Gene	Primers	PCR Conditions	Product Length
Ezh2	Forward: 5'- CCCATGTTTAAGGGCATAGTG ACATG-3'<br>Reverse1: 5'-TCGAGGGACCTAATAACTCGT ATAGCA-3'<br>Reverse2: 5'-ATGTGCAGGTCAGTCAGCAAC TTCAG-3'	35 cycles:<br>94°C-30s<br>61°C-30s<br>72°C-60s	WT allele: 410 bp<br>Floxed allele: 450 bp
Isl1-cre	Forward: 5'-GCCACTATTTGCCACCTAGC-3'<br>Reverse: 5'-AGGCAAATTTTGGTGTACGG-3'	38 cycles:<br>94°C-30s<br>61°C-30s<br>72°C-60s	Transgene fragment: 250 bp
ROSA26^{mTmG}	Forward: 5'-CTCTGCTGCCTCCTGGCTTCT-3'<br>Reverse1: 5'-CGAGGCGGATCACAAGCAATA-3'<br>Reverse2: 5'-TCA ATG GGC GGG GGT CGT -3'	39 cycles:<br>94°C-30s<br>64°C-30s<br>72°C-60s	WT allele: 330 bp<br>Mutant allele: 250 bp

Biology Open • Supplementary information
